# Hydralazine Use and Risk of Vasculitis

**DOI:** 10.1001/jamanetworkopen.2026.1943

**Published:** 2026-03-16

**Authors:** Deena Fremont, Shan Dhaliwal, Mark Canney, Ayub Akbari, Gregory L. Hundemer, Vimal K. Derebail, Manish M. Sood, David Massicotte-Azarniouch

**Affiliations:** 1 Ottawa Hospital Research Institute, Ottawa, Ontario, Canada; 2 ICES uOttawa, Ottawa, Ontario, Canada; 3 Division of Nephrology, Department of Medicine, University of Ottawa, Ottawa, Ontario, Canada; 4 UNC Kidney Center, Division of Nephrology and Hypertension, University of North Carolina at Chapel Hill, Chapel Hill

## Abstract

**Importance:**

Hydralazine is a selectively used cardiovascular medication with case reports and case series demonstrating an association between the use of hydralazine and antineutrophil cytoplasmic antibody–associated vasculitis.

**Objective:**

To assess the risk of vasculitis associated with hydralazine use compared with use of an angiotensin-converting enzyme inhibitor (ACE) or angiotensin receptor blocker (ARB).

**Design, Setting, and Participants:**

This population-based, retrospective cohort study assessed adults 66 years or older from Ontario, Canada, who were newly prescribed hydralazine from January 1, 2008, to December 31, 2021. Data analysis was performed from May to August 2025.

**Exposure:**

Newly dispensed hydralazine in an outpatient setting.

**Main Outcomes and Measures:**

The main outcome was a diagnosis of vasculitis using *International Statistical Classification of Diseases and Related Health Problems, Tenth Revision (ICD-10) *codes. Overlap-weighted Cox proportional hazards regression was used to examine the association between hydralazine use (active comparator: ACE or ARB) with new-onset vasculitis.

**Results:**

Of a total of 583 136 eligible adults (mean [SD] age, 73.0 [7.2] years; 51 827 [55.2%] female), 40 748 individuals were dispensed hydralazine and 542 388 an ACE or ARB. Hydralazine use compared with ACE or ARB use was associated with a higher risk of vasculitis during follow-up (hydralazine: 328 events [0.8%]; ACE or ARB: 2712 events [0.5%]; absolute risk difference, 0.3 percentage points; hazard ratio, 1.19; 95% CI, 1.04-1.37). Results were no longer significant when accounting for the competing risk of death.

**Conclusions and Relevance:**

In this cohort study of adults who were newly prescribed hydralazine, vasculitis associated with hydralazine was rare, despite multiple reported cases. Use of hydralazine is unlikely to be associated with a clinically meaningful increased risk of vasculitis.

## Key Points

**Question:**

What is the risk of vasculitis associated with hydralazine use compared with use of an angiotensin-converting enzyme inhibitor (ACE) or angiotensin receptor blocker (ARB)?

**Findings:**

This cohort study of 583 136 adults in Ontario, Canada, found that, when compared with ACE or ARB use, hydralazine was associated with a higher risk of vasculitis during follow-up. However, vasculitis events were rare in both groups.

**Meaning:**

This study suggests that vasculitis associated with hydralazine is a rare event and use is unlikely to be associated with a clinically meaningful increased risk of vasculitis.

## Teaser

This cohort study of Canadian adults examines whether hydralazine use is associated with a higher risk of vasculitis compared with angiotensin-converting enzyme inhibitor or angiotensin receptor blocker use.

## Introduction

Hydralazine is a rapid, direct-acting vasodilator first introduced in the 1950s.^1^ It remains routinely prescribed for cardiovascular conditions due to its widespread availability and low cost.^2^ In heart failure, hydralazine (when coupled with isosorbide dinitrate) is a class 2b, level B guideline recommended therapy with recent concerns of underuse in select populations.^2,3^ It is further used as a third- or fourth-line agent in treatment of hypertension, often in select scenarios, such as pregnancy or hypertensive emergencies.

Reports of autoimmunogenic activity with hydralazine use first emerged in the 1980s with case reports of drug-induced lupus, cutaneous leukocytoclastic vasculitis, and antineutrophil cytoplasmic antibody (ANCA)–associated vasculitis (AAV).^4^ Hydralazine-associated vasculitis may present with pulmonary-renal syndrome, rapidly progressive glomerulonephritis, and permanent kidney or lung damage.^5,6^ As such, the product monograph warns of autoimmune disease as a potential adverse event and recommends periodic routine monitoring for a complete blood cell count, antinuclear factors, and urinary abnormalities. Clinical trials^7,8,9^ report only one case of lupuslike syndrome in more than 700 individuals randomized to hydralazine. Despite their inherent potential biases, health administrative databases are a useful method to examine rare adverse drug events.^10^ To date, the true extent of autoimmune-related adverse events, such as AAV, with hydralazine use remains uncertain.

We examine the population-level incidence of hydralazine-associated AAV compared with a commonly prescribed class of comparable medications, angiotensin-converting enzyme inhibitor (ACE) or angiotensin receptor blocker (ARB). We hypothesize that hydralazine use would be associated with a higher risk of vasculitis compared with ACE or ARB use.

## Methods

### Study Design and Setting

We conducted a population-based, retrospective cohort study using health administrative databases available at ICES (formerly known as the Institute for Clinical Evaluative Sciences). ICES holds routinely collected and individually linked health administrative data collected through the delivery of services covered by the province of Ontario’s universal single-payer health care system. Ontario is Canada’s largest province, with a population of more than 16 million residents, of which 18% are older than 65 years.^11^ The use of the data in this project is authorized under section 45 of Ontario’s Personal Health Information Protection Act and does not require review by a research ethics board or participant informed consent. The reporting of this study follows the Strengthening the Reporting of Observational Studies in Epidemiology (STROBE) guidelines for cohort studies.^12^

### Data Sources

Baseline characteristics, medication use, and outcome data were ascertained from ICES databases. The Registered Persons Database was used for demographics and vital status. The Discharge Abstract Database and the National Ambulatory Care Reporting System contain information and diagnostic codes on all acute care hospitalization and emergency department visits, respectively, and were used to obtain the relevant data. Information on baseline laboratory values was obtained from the Ontario Laboratory Information System, which captures 95% of laboratory tests in Ontario. Medication information was obtained from the Ontario Drug Benefit (ODB), which contains highly accurate records of outpatient prescription claims, with an error rate less than 1%.^13^ These datasets were linked using unique encoded identifiers and analyzed at ICES. Where possible, we used validated codes to identify baseline characteristics (eMethods in Supplement 1).

### Study Cohort

The study population included all Ontario residents 66 years or older between January 1, 2008, and December 31, 2021, who received a new outpatient prescription for hydralazine vs ACE or ARB. All Ontario residents older than 65 years have prescription medications covered through ODB. Although ODB eligibility begins at 65 years of age, the minimum age of 66 years was selected to allow a 1-year lookback period for medication use to capture incident drug use. Individuals receiving a new hydralazine prescription were compared with individuals receiving a new prescription of an ACE or ARB (new user active comparator study design^14^). ACEs and ARBs are often prescribed for similar clinical indications as hydralazine, such as hypertension or heart failure. We excluded the following individuals: (1) those older than 105 years at the index date, (2) non-Ontario residents or non–Ontario Health Insurance Plan eligible individuals, (3) those with prior hydralazine use, (4) those with prior vasculitis diagnosis, and (5) those with prior kidney transplant.

The date of first hydralazine dispensing served as the index date for the exposure group, and the date of first ACE or ARB dispensing served as the index date for the control group. Censoring occurred at the first study outcome event, death, emigration from province, or end of follow-up (end of study period: December 31, 2022).

### Outcomes

The outcome of interest was a new diagnosis of vasculitis at any point after hydralazine use. Vasculitis was identified using any *International Statistical Classification of Diseases and Related Health Problems, Tenth Revision (ICD-10) *diagnosis code for vasculitis (eMethods in Supplement 1). The *ICD-10* codes have been in use since 2002 in Ontario, Canada. Administrative data to identify vasculitis are susceptible to misclassification or miscoding. As such, we used a broad approach to capture any possible vasculitis by using any vasculitis code. We also examined the specific AAV *ICD-10* diagnosis codes (M313, M3131, M317, M301). A previous Canadian study^15^ examined the use of a single diagnostic code for identifying vasculitis, with a reported sensitivity of 52.6% and specificity of 93.1%.

### Statistical Analysis

We used overlap propensity score weighting to create balance between the study groups (hydralazine vs ACE or ARB).^16^ A propensity score for hydralazine exposure was calculated followed by overlap-weighted analyses to create a balanced cohort between the exposure and control groups in terms of 21 baseline variables included to calculate the propensity score.^16,17^ Baseline variables were chosen based on availability from data sources and on potential association with exposure or outcome. These variables included age at index, sex, median household income quintile associated with residence, postal code–based rurality, year of index date, estimated glomerular filtration rate (eGFR) (using serum creatinine level closest to the index date within 1 year), any specialist visit in the prior 5 years (rheumatologist or nephrologist), comorbidities (arrhythmia, cancer, chronic obstructive pulmonary disease, diabetes, hypertension, ischemic stroke, myocardial infraction, peripheral vascular disease, coronary artery disease, deep vein thrombosis, coronary artery bypass graft, or congestive heart failure), and history of medication use within 1 year of the index date (allopurinol, antihypertensive agent, methimazole, prednisone, propylthiouracil, and statins). Restricted cubic splines were applied to both age and eGFR.

Standardized differences were used to examine differences between the study groups after the overlap weighting procedure.^18^ We calculated the crude counts, cumulative incidences, and weighted Cox proportional hazards models to examine the association between hydralazine use and vasculitis diagnosis. No adjustments were performed for multiple testing, so positive associations should be interpreted cautiously due to the risk for type I error. Analysis was conducted from May to August 2025 using SAS, version 8.3 (SAS Institute Inc). A 2-sided *P* < .05 was considered statistically significant.

We performed the following additional analyses. First, we analyzed the association with vasculitis diagnosis, accounting for the competing risk of death using Fine-Gray subdistribution hazards ratios (HRs). Second, we analyzed the association between hydralazine use and a first positive ANCA serologic test results. Third, we analyzed the association between hydralazine use and vasculitis diagnosis when censoring at hydralazine discontinuation (defined as at least 90 days without dispensing from the end of last prescription end date). Fourth, we examined the association of vasculitis with initial hydralazine dose; for this analysis, we limited the cohort to hydralazine users only with the exposure being median dose or lower compared with greater than the median dose. Fifth, we examined the association between hydralazine use and vasculitis diagnosis using a new α-blocker prescription as a control group because this represents a fourth- or fifth-line blood pressure–lowering agent similar to hydralazine. Sixth, we examined the association between hydralazine use and vasculitis diagnosis occurring after a latency period of 90, 180, and 365 days to account any potential delay onset of vasculitis.

## Results

### Study Cohort Characteristics

**Figure 1.**
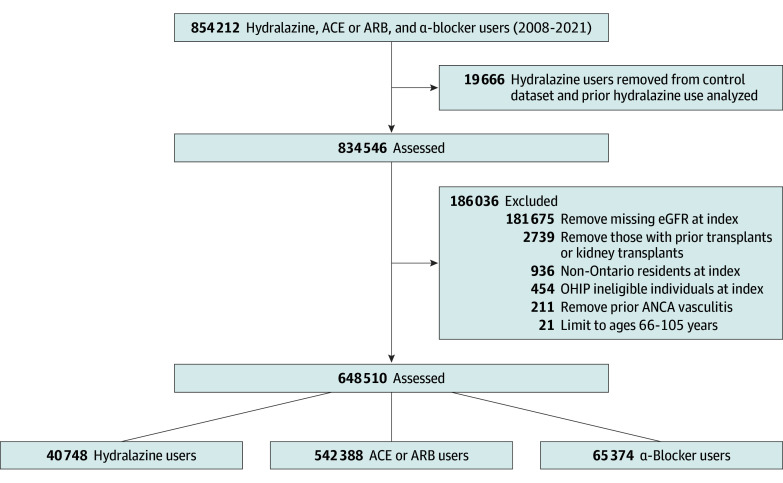
Cohort Flow Diagram ACE indicates angiotensin-converting enzyme inhibitor; ANCA, antineutrophil cytoplasmic antibody; ARB, angiotensin receptor blocker; eGFR, estimated glomerular filtration rate; OHIP, Ontario Health Insurance Plan.

During the study period, 583 136 eligible adults (mean [SD] age, 73.0 [7.2] years; 51 827 [55.2%] female), including 40 748 new hydralazine users and 542 388 new ACE or ARB users, were identified (Figure 1). The mean (SD) study follow-up was 5.9 (1.7) years. Compared with ACE or ARB users, hydralazine users were older, had more recent index date year, and had more prior nephrology and rheumatology specialist visits. After overlap-weighted propensity score weighting, no significant differences were found between the 2 groups in baseline characteristics (Table 1).

**Table 1.** Baseline Characteristics of Hydralazine and ACE or ARB Medication Users

Characteristic	No. (%) of participants^a^		Standardized difference (before weighting)^b^
	Hydralazine (n = 40 748)	ACE or ARB (n = 542 388)	
Age at index, mean (SD), y	79.0 (7.8)	74.1 (7.0)	0.66
Rural residence	3415 (8.4)	69 052 (12.7)	0.14
Sex			
Female	22 000 (54.0)	299 827 (55.3)	0.03
Male	18 748 (46.0)	242 561 (44.7)	0.03
Income quintiles			
First (lowest)	9718 (23.8)	106 607 (19.7)	[Reference]
Second	8979 (22.0)	112 650 (20.8)	0.03
Third	8124 (19.9)	108 500 (20.0)	0.002
Fourth	7407 (18.2)	106 464 (19.6)	0.04
Fifth (highest)	6352 (15.6)	106 623 (19.7)	0.12
Missing	168 (0.4)	1544 (0.3)	0.02
Index year			
2008	448 (1.1)	15 500 (2.9)	0.13
2009	1064 (2.6)	31 200 (5.8)	0.16
2010	1391 (3.4)	38 464 (7.1)	0.16
2011	1572 (3.9)	37 620 (6.9)	0.14
2012	2273 (5.6)	37 863 (7.0)	0.06
2013	2390 (5.9)	39 867 (7.4)	0.06
2014	2718 (6.7)	39 811 (7.3)	0.02
2015	3010 (7.4)	39 453 (7.3)	0.004
2016	3320 (8.1)	42 157 (7.8)	0.01
2017	3940 (9.7)	43 960 (8.1)	0.06
2018	4462 (11.0)	46 755 (8.6)	0.08
2019	4738 (11.6)	47 694 (8.8)	0.09
2020	4377 (10.7)	37 175 (6.9)	0.14
2021	5045 (12.4)	44 869 (8.3)	[Reference]
Specialist visits within 5 y of index date			
Nephrology visits			
Mean (SD)	7.9 (17.1)	0.6 (4.1)	1.00
No. (%)	21 189 (52.0)	50 984 (9.4)	1.04
Rheumatology visits			
Mean (SD)	13.06 (14.56)	4.86 (8.59)	0.66
No. (%)	37 078 (91.6)	352 010 (64.9)	0.68
Inpatient eGFR, mean (SD), mL/min/1.73 m^2^	87.68 (4.20)	90.51 (3.93)	0.70
Comorbidities within 5 y of index date			
Arrhythmia	6353 (15.6)	26 192 (4.8)	0.36
Cancer	2159 (5.3)	19 224 (3.5)	0.08
Chronic obstructive pulmonary disease	5198 (12.8)	34 923 (6.4)	0.22
Diabetes	3552 (8.7)	61 365 (11.3)	0.09
Hypertension	3431 (8.4)	155 032 (28.6)	0.54
Ischemic stroke	3018 (7.4)	19 063 (3.5)	0.17
Myocardial infraction	3998 (9.8)	28 152 (5.2)	0.18
Peripheral vascular disease	1465 (3.6)	4637 (0.9)	0.19
Coronary artery disease	18 289 (44.9)	114 726 (21.2)	0.52
Deep vein thrombosis	1259 (3.1)	5491 (1.0)	0.15
Coronary artery bypass graft	1045 (2.6)	7838 (1.4)	0.08
Congestive heart failure	11 998 (29.4)	36 193 (6.7)	0.62
History of medication use			
Allopurinol	5655 (13.9)	15 791 (2.9)	0.40
Antihypertensive agent	39 567 (97.1)	298 886 (55.1)	1.13
Methimazole	79 (0.2)	501 (0.1)	0.03
Prednisone	4960 (12.2)	31 286 (5.8)	0.23
Propylthiouracil	27 (0.1)	278 (0.1)	0.006
Statin	29 669 (72.8)	283 371 (52.2)	0.44

Abbreviations: ACE, angiotensin-converting enzyme inhibitor; ARB, angiotensin receptor blocker; eGFR, estimated glomerular filtration rate.

^a^ Unless otherwise indicated.

^b^ Standardized difference (after weighting) is 0 for all variables.

### Vasculitis Diagnosis

**Figure 2.**
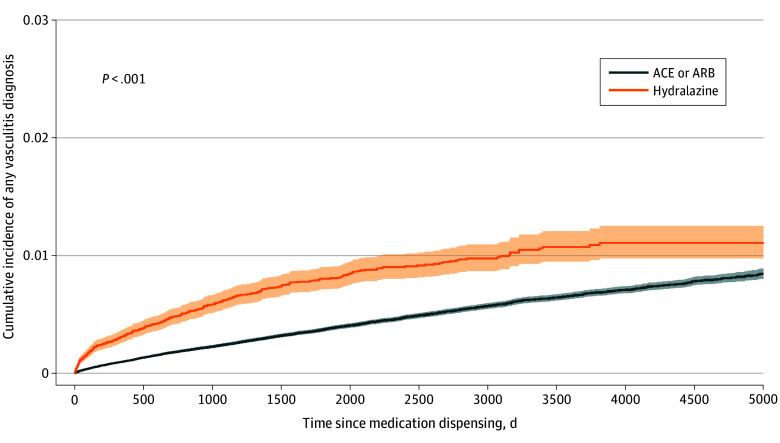
Cumulative Incidence Plot for Any Vasculitis ACE indicates angiotensin-converting enzyme inhibitor; ARB, angiotensin receptor blocker.

For any vasculitis code, in hydralazine users compared with ACE or ARB users, there were 328 (absolute risk, 0.8%; crude incidence rate, 234.7 per 100 000 person-years) vs 2712 (absolute risk, 0.5%; incidence rate, 81.6 per 100 000 person-years) vasculitis diagnoses after a first outpatient dispensing of medication (absolute risk difference, 0.3 percentage points), occurring after a median (IQR) 545 (129-1150) days vs 1200 (463-2222) days of dispensing, respectively (Table 2 and Figure 2). The overlap propensity score–weighted HR for any vasculitis diagnosis in hydralazine users was 1.19 (95% CI, 1.04-1.37) compared with ACE or ARB users (Table 2). In the whole study cohort, there were fewer than 6 diagnoses of AAV (using specific AAV codes) in hydralazine users (event number too small to calculate incidence rate or HR) compared with 60 in ACE or ARB users (<0.1%).

**Table 2.** Risk of Any Vasculitis With Hydralazine vs ACE or ARB

Characteristic	No. of events/total No. of patients	Absolute risk, %	Crude incidence rate (100 000 patient-years)	Overlap propensity score weighted, HR (95% CI)
Hydralazine	328/40 748	0.8	234.7	1.19 (1.04-1.37)
ACE or ARB	2712/542 388	0.5	81.6	1 [Reference]

Abbreviations: ACE, angiotensin-converting enzyme inhibitor; ARB, angiotensin receptor blocker; HR, hazard ratio.

### Additional Analyses

When accounting for the competing risk of death, the subdistribution HR for vasculitis diagnosis with hydralazine use was 1.01 (95% CI, 0.88-1.16) compared with ACE or ARB use (Table 3). When examining the outcome of positive ANCA serologic test results after drug exposure, there were only 17 positive ANCA serologic test results in new hydralazine users compared with 80 in ACE or ARB users. Due to the small number of events, no further tests were performed. When censoring at discontinuation of exposure medication, for hydralazine users vs ACE or ARB users, the HR for any vasculitis diagnosis was 1.36 (95% CI, 1.12-1.67) (Table 3). When examining hydralazine dose, the median daily dose was 40 mg, and the HR for any vasculitis diagnosis with a higher dose (>40 mg) of hydralazine was 1.28 (95% CI, 1.03-1.60) compared with a lower dose (<40 mg). When comparing hydralazine users with α-blocker users, the HR for any vasculitis was 1.16 (95% CI, 0.98-1.37) (Table 3). When using a latency period of 90, 180, or 365 days, the HRs were 1.10 (95% CI, 0.94-1.28), 1.10 (95% CI, 0.94-1.29), and 1.12 (95% CI, 0.94-1.33), respectively.

**Table 3.** Vasculitis Diagnoses in the Study Participants by Analysis Group

Analysis group	No./total No. (%) of participants with vasculitis diagnoses
Competing risk of death	
Hydralazine^a^	328/40 748 (0.8)
ACE or ARB^b^	2712/542 388 (0.5)
Censoring at discontinuation	
Hydralazine	159/40 748 (0.4)
ACE or ARB	1413/542 388 (0.3)
Higher hydralazine dose	
High dose	181/19 709 (0.9)
Low dose	147/21 039 (0.7)
Risk of vasculitis	
Hydralazine	328/40 748 (0.8)
α-Blocker	447/65 372 (0.7)

Abbreviations: ACE, angiotensin-converting enzyme inhibitor; ARB, angiotensin receptor blocker.

^a^ There were 22 493 deaths in this group.

^b^ There were 136 644 deaths in this group.

## Discussion

In this retrospective cohort study among adults older than 65 years, we found 40 748 new hydralazine users during a 14-year period. We examined the occurrence of any vasculitis diagnosis using *ICD-10* codes and found an increased risk of 20%, with an absolute risk of 1 additional case of vasculitis leading to a hospital encounter for every 333 new hydralazine users compared with new ACE or ARB users. This risk was attenuated and no longer significant when accounting for the competing risk of death and when comparing with α-blockers. Our results suggest new hydralazine use in older adults is rare and may be associated with at most a modest increased risk, which may not be clinically meaningful when considering risks and benefits of hydralazine use.

There is limited large-scale clinical evidence examining the association between hydralazine use and the occurrence of vasculitis in routine clinical care. Many case reports and case series have described possible hydralazine-associated AAV.^19,20,21,22^ Experimental research^23^ proposes a mechanistic pathway, whereby hydralazine may lead to conformational changes in myeloperoxidase, altering its pathogenicity and subsequently leading to the production of autoantibodies. Despite the multitude of reports and mechanistic plausibility, it is difficult to ascertain causality. AAV is a rare diagnosis and AAV caused by hydralazine even rarer because it is not a routinely used outpatient medication. This poses problems when trying to examine associations with such a rare event. We found low rates of subsequent AAV diagnosis identified through *ICD-10* codes among hydralazine users (<6 diagnoses). This low number of events precluded our ability to examine incidence rates and risks compared with ACE and ARB users. One previous case series described the features of hydralazine-associated AAV using *International Classification of Diseases, Ninth Revision (ICD-9) *and *ICD-10* codes with a positive ANCA laboratory test results during a 15-year period. There were 323 cases of AAV identified, and only 12 individuals were previously exposed to hydralazine at the time of diagnosis.^5^ This finding highlights the difficulty in properly assessing and quantifying the risk of AAV associated with hydralazine, even when using health administrative databases, which cover a population of more than 16 million people.

We attempted to quantify the incidence and risk of AAV after hydralazine use, while ensuring a temporal relation with diagnosis occurring after a first exposure to hydralazine. There were too few events of new occurrence of a specific AAV *ICD-10* diagnostic code to make meaningful estimations. Even when examining the occurrence of a positive ANCA serologic test result after hydralazine use, there were too few events. We expanded our outcome to any vasculitis diagnosis, acknowledging the loss of specificity but the potential increase in sensitivity to identify individuals with vasculitis induced by hydralazine. Hydralazine has been associated with lupuslike manifestations,^24^ and AAV associated with hydralazine may have atypical features of otherwise classic, idiopathic AAV.^25^ Therefore, cases of new vasculitis potentially caused by hydralazine may be described in patient medical records and discharge summaries in various ways, so it may not always be captured with one of the specific AAV *ICD-10* diagnostic codes. Even when using a much less specific diagnostic capture method, there was only a mildly elevated risk of vasculitis associated with hydralazine compared with an ACE or ARB. This association was lost when accounting for the competing risk of death, an important consideration in an older population,^26^ and when comparing with α-blockers. Therefore, based on our data, it is unclear whether hydralazine is significantly associated with AAV. This does not rule out the possibility of cases in which hydralazine may indeed lead to AAV. However, this factor must be considered when assessing the frequency of hydralazine use and AAV. Although clinicians considering hydralazine to treat a patient may worry about inducing organ-threatening vasculitis, in absolute terms this would be an unlikely event. Currently, hydralazine drug monographs call for routine clinical and laboratory monitoring of vasculitis and lupuslike syndromes, with some mentioning that risk occurs with duration of drug use.^27,28,29,30,31^ Our findings allow for product monographs to be updated with quantitative risk estimates and facilitate risk-benefit decision-making. Hydralazine (combined with isosorbide dinitrate) is a well-established, mortality-reducing therapy for heart failure, especially in populations that are ACE and ARB resistant.^3^ The fear of vasculitis should not necessarily deter clinicians from using hydralazine if otherwise appropriate.

### Strengths and Limitations

This study has strengths. We present a large study in which we sought to quantify the risk and incidence of hydralazine-associated vasculitis, something for which there are few data available. Obtaining accurate risk estimates using large administrative datasets is challenging; however, this must be weighed against the strengths of comprehensive, population-based coverage of outcomes and accurate drug exposure.

Our study also has limitations that warrant consideration. First, the use of *ICD-10* diagnostic codes to identify events of vasculitis means we could only identify cases diagnosed through a hospital encounter. This would have limited our ability to detect events diagnosed and managed solely on an outpatient basis. That being said, most reports^20,21,22^ of hydralazine-induced vasculitis describe organ-threatening manifestations, which would likely lead to a hospital encounter. Second, there is the risk of misclassification and false-negative results with the use of *ICD-10* codes to capture diagnoses, which could also have limited the number of events detected. We used a broad definition of vasculitis that would be more sensitive and less specific (a more conservative approach from a public health perspective), acknowledging the outcomes detected may not exclusively represent hydralazine-induced vasculitis. Our findings may represent overestimates. This means the true risk estimate could be even closer to the null, which would not change our conclusions. Third, our study population was limited to those older than 65 years to capture medication dispensing. This affects generalizability and contributes to a lower number of events, although previous research^21^ reports a mean age of 69 years, so most cases should have been included and there would be no reason to believe hydralazine-induced vasculitis risk would differ between older and younger adults. Fourth, because our exposure was medication dispensing in the outpatient setting, we would not capture cases of vasculitis due to hydralazine used solely during a hospital encounter. Overall, some of these limitations may lead to missed events, which could reduce the precision of our estimates. Fifth, there are case reports of relatively minor drug-induced leukocytoclastic vasculitis with ACE or ARB use that may have contributed to a higher incidence with ACE or ARB users.^32^ Sixth, we found similar risk estimates when using α-blockers as the control group, which like hydralazine may be used as fourth- or fifth-line antihypertensives. However, this cannot fully account for unmeasured confounding. For example, we lacked information on frailty and race that may influence prescription and identification of outcomes.

## Conclusions

In this cohort study of adults who were newly prescribed hydralazine, the use of hydralazine may be associated with a small measurable risk of vasculitis compared with ACE or ARB use; however, this finding did not appear to represent a clinically meaningful risk given the rarity of the disease. Although our findings did not determine whether hydralazine caused vasculitis, concern for vasculitis should probably not be the main factor driving a clinician’s decision on the appropriateness of using hydralazine for their patient.

## Notes

Supplement 1. **eMethods. **Supplementary Material

Supplement 2.
Data Sharing Statement
