# Supplementary material for: Hydralazine Use and Risk of Vasculitis
Source: JAMA Netw Open. 2026 Mar 16;9(3):e261943. doi: 10.1001/jamanetworkopen.2026.1943 (PMC12993695; doi:10.1001/jamanetworkopen.2026.1943)
Supplement: Supplement 1. — eMethods. Supplementary Material [file jamanetwopen-e261943-s001.pdf]

Supplemental Online Content

Fremont D, Dhaliwal S, Canney M, et al. Hydralazine use and risk of vasculitis. *JAMA Netw Open*. 2026;9(3):e261943. doi:10.1001/jamanetworkopen.2026.1943

eMethods. Supplementary Material

This supplemental material has been provided by the authors to give readers additional information about their work.

© 2026 Fremont D et al. *JAMA Network Open*.

eMethods. Supplementary Material

Data Sources, Information, and Linked Datasets Used in Study

ICES databases and associated descriptions used to make the cohort.

ICES Database	Description
Discharge Abstract Database (DAD)	Contains demographic, diagnostic, and treatment information for all acute care hospitalizations
National Ambulatory Care Reporting System (NACRS)	Contains demographic, diagnostic, and treatment information for hospital and community-based ambulatory care. This dataset includes both outpatient and community-based clinics and emergency departments.
ODB (Ontario Drug Benefit)	Captures all medications covered by the provincial health insurance prescribed in the community
Ontario Health Insurance Plan (OHIP)	Contains records of claims for physician services covered by the provincial government
Registered Persons Database (RPDB)	Demographic and vital statistics information for those eligible for insurance health services in Ontario, Canada
Drug Identification Number (DIN)	Contains information pertaining to unique numbers assigned to all drugs in Canada. The DIN databases identify all drug products, including manufacturer, product name, active ingredient(s), strength(s) of active ingredient(s), pharmaceutical form, and route of administration.
Postal Code Conversion File (PCCF)	Contains information on the rurality (urban vs. rural) of primary residence address
Ontario Laboratory Information System (OLIS)	Contains laboratory records of Public Health Ontario, hospital, and community laboratories
**ICES Derived Cohorts**	
Congestive Heart Failure (CHF)	Validated registry of all individuals in Ontario with associated diagnosed condition
Chronic Obstructive Pulmonary Disease (COPD)	
Hypertension (HYPER)	
Ontario Diabetes Dataset (ODD)	

© 2026 Fremont D et al. *JAMA Network Open*.

Ontario Myocardial Infraction Dataset (OMID)	
Candain Organ Replacement Register (CORR)	

© 2026 Fremont D et al. *JAMA Network Open*.

Cohort: Code Description

Inclusion/Exclusion Criteria	Data Sources	Variables	Time
Inclusion Criteria			
Pull all hydralazine/ACEi/ARB/Alpha-blocker drugs dispensed	DIN<br>ODB	Hydralazine DINS: 00005525, 00005533, 00005541, 00441619, 00441627, 00441635, 00759473, 00759481, 01913204, 02457865, 02457873, 02457881, 02468778, 02468786, 02468794<br><br>Alpha-blockers: drugname in (“DOXAZOSIN”, “PRAZOSIN”, “TERAZOSIN”)<br><br>To find ACEi/ARB:<br>Subclnam = ACE INHIBITORS, ACE INHIBITORS COMBINATION, ANGIOTENSIN II ANTAGONIST<br><br>SERVDATE	1 January 2008 – 31 December 2021
Exclusion Criteria			
Data cleaning<br>a. Missing or invalid IKN<br>b. Missing or invalid age (>105)<br>c. Missing or invalid sex<br>d. Death on or before index date<br>e. Non-Ontario resident<br>f. Not OHIP eligible at index date	RPDB	VALIKN<br>AGE<br>SEX<br>DTHDATE<br>PRCDDABLK	Reference date=index date
Age < 66	RPDB	AGE	Reference date=index date
Previous ANCA Vasculitis diagnosis	DAD<br>NACRS	ICD-10: M313 M313.1 M317 M301	Lookback 5-years from index date

© 2026 Fremont D et al. *JAMA Network Open*.

Prior dispensing of hydralazine	ODB	DIN: 00005525, 00005533, 00005541, 00441619, 00441627, 00441635, 00759473, 00759481, 01913204, 02457865, 02457873, 02457881, 02468778, 02468786,<br>02468794	Lookback 1-year from index date
End-stage kidney disease	OLIS<br><br>CORR	Two outpatient eGFR measures within 5% of each other where eGFR < 15<br><br>Detection of any 2 outpatient claims for dialysis using data over a 5-year period: Treatment_code /= to 171, 181	
Prior kidney transplant recipients	CORR<br><br>DAD<br>OHIP	Treatment code = 171, 181 and transplanted organ type code1-3: 10, 11, 12, 18 or 19<br><br>Feecode: S435, S434<br>CCI 1PC85	On or before index date

Baseline Characteristics: Code Descriptions

Baseline Characteristic	Data Source(s)	Variable/Corresponding code	Time	Reporting Detail
Age	RPDB	BDATE	Reference date = index date	Mean (SD) Median (IQR)
Sex	RPDB	SEX	Reference date=index date	N (%) - Female
Rural status	RPDB	RURAL	Reference date = index date	N (%) – rural, urban, missing
Year of index	ODB	SERVDATE		N (%) by year<br>2008 - 2021

© 2026 Fremont D et al. *JAMA Network Open*.

# of hospitalizations	DAD	Admdate	3 years prior to index date	Mean (SD) Median (IQR)
# of nephrologist encounters	OHIP	Nephrology consultation: A165 1765 A160 A865 A166 A163 A164 A161 A168<br><br>Non-emergency nephrology visit: C165 C765 C160 C865 C166 C163 C164 C161<br><br>Nephrology subsequent visits: C162 C167 C169<br><br>Non-emergency LTC in-patient services: w165, W765, W160, W865, W166, W862, W864, W867, W869, W164, W162, W161, W982, W163, W168, W972, W121	5 years prior to or on index date	Mean (SD) Median (IQR)
# of rheumatologist encounters	OHIP	Rheumatology consultations: A485 A765 A590 A595 A486 A483 A484 A481 A488 A480 K481 K480<br><br>Non-emergency hospital visit: C485 C765 C590 C595 C486 C483 C484 C481 C480 C<br><br>Subsequent visits: C482 C487 C489<br><br>Non-emergency hospital in-patient services: C355, C935, C356, C353, C354, C352, C357, C359, C122, C123, C124<br><br>Subs. Visits by MRP following transfer from an Intensive care area: C142, C143, C121, C358, C982 (Pall. Care)<br><br>Non-emergency long-term care in-patient services: W355, W356,	5 years prior to or on index date	Mean (SD) Median (IQR)

© 2026 Fremont D et al. *JAMA Network Open*.

Serum Creatinine	OLIS	Observation code: 14682-9	Most recent within 1-year prior to index date	Mean (SD)<br>Median (IQR)
Estimated glomerular filtration rate	OLIS	Observation code: 14682-9	Most recent within 1-year prior to index date	Mean (SD)<br>Median (IQR)
Diabetes Mellitus	ODD	Diagdate	5-year lookback window from index date	N (%)
Hypertension	HYPER	Diagdate	5-year lookback window from index date	N (%)
Ischemic stroke	DAD<br>NACRS	ICD-10: H341 I630 I631 I632 I633 I634 I635 I638 I639 I64	5-year lookback window from index	N (%)
Myocardial Infarction	DAD<br>NACRS<br>OMID	ICD 10: I21 I22<br><br>diagdate	5-year lookback window from index	N (%)
Arrhythmia	DAD<br>NACRS<br>OHIP	ICD 10: "I48", "I44", "I45", "I47", "I4900", "I4901", "I491", "I492", "I493", "I494", "I498", "I499", "R000" "R001"<br><br>OHIP feecodes: "G178", "G179", "G249", "G261", "G259", "Z443", "Z431", "Z437"	5-YEARS ON OR BEFORE INDEX	N (%)
Congestive Heart Failure	CHF<br>DAD<br>NACRS	Diag date	5-year lookback window from index	N (%)

	OHIP	ICD-10: I099 I420 I425 I426 I427 I428 I429 I43 I500 I501 I509 I255 J81<br><br>CCI: 1HP53 1HP55 1HZ53GRFR 1HZ53LAFR 1HZ53SYFR<br><br>OHIP Feecode: R702 Z429		
Coronary Artery Disease	DAD<br>NACRS<br>OHIP	ICD-10: I21 I22 Z955 T822<br><br>CCI: 1IJ50 1IJ76<br><br>Feecode: R741 R742 R743 G298 E646 E651 E652 E654 E655 Z434 Z448	5-yeear lookback window from index	N (%)
Coronary Artery Bypass Graft	DAD<br>OHIP	CCI: 1IJ76<br><br>Feecode: R742 R743 E654 E645 E652 E646	5-year lookback window from index	N (%)
Peripheral Vascular Disease	DAD<br>NACRS<br>OHIP	ICD 10: "I700", "I702", "I708", "I709", "I731", "I738", "I739", "K551"<br><br>CCI: "1KA76", "1KA50", "1KE76", "1KG50", "1KG57", "1KG76MI", "1KG87", "1IA87LA", "1IB87LA", "1IC87LA", "1ID87", "1KA87LA", "1KE57"<br><br>OHIP fee codes: "R787", "R780", "R797", "R804", "R809", "R875", "R815", "R936", "R783", "R784","R785", "E626", "R814", "R786", "R937", "R860", "R861", "R855", "R856", "R933", "R934", "R791", "E672", "R794", "R813", "R867", "E649"	5-year lookback window from index	N (%)

© 2026 Fremont D et al. *JAMA Network Open*.

Venous thromboembolism (deep vein thrombosis or pulmonary embolism)	DAD<br>NACRS<br>OHIP	Algorithm for Venous thromboembolism:<br><br>Either of the following (limit to first occurrence):<br>A. A combination of one of the following OHIP dxcodes & feecodes within 7 days of each other (servdate):<br>• dxcode in: 451, 671, 415, 677 AND<br>• feecode in: “J198” “J498” “J193” “J493” “J202” “J502” J659, J660, J859, J860, X406, X407, X125<br>• If intnx(‘day’,servdate_dxcode,-7,’s’) <= servdate_feecode <= intnx(‘day’,servdate_dxcode,7,’s’) then VTE (feecode within 7 days before or after diagnosis code)<br><br>OR<br>B. one of dx10code (type=any) or an incodein a hospitalization or ED record .<br>• Dx10code in: "I80.1", "I80.2", "I80.3", I82.2, I82.8, I82.9, O87.1, O87.8, O87.9, I26, O88.2;<br>Incodes: "3KX30DA" "3KX30DB" "3KX30DC" "3KX30DD" "3KR10VA" "3KR10VC" "3KR10VN" "3KR12VA" "3KX10VA" "3KX10VC" "3KX10VN" "3KX10VX" "3KX12VA" "3IM10VC" "3IM10VX" "3IM10VY" "3IM12VA" "3GT70CA" "3GT70CC" "3GT70CE" "3GT70KC" "3GT70KD" '3GT70KE" "3JY10VA" "3JY10VC" "3JY10VN" "3JY10VX" "3JY12VA" "3JY20WC" "3JY20WE" "3GT20WC" "3GT20WE")	5-year lookback window from index	N (%)
Chronic Obstructive Pulmonary Disease	DAD<br>COPD	ICD-10: J41 J43 J44<br><br>DIAGDATE	5-year lookback window from index	N (%)
Major Cancer	DAD<br>NACRS	ICD: C00-C97	5-year lookback window from index	N (%)
Prednisone	ODB	If drugname in “PREDNISONE”	1-year lookback window from index	N (%)

© 2026 Fremont D et al. *JAMA Network Open*.

Anti-hypertensive agent<br>- Beta-blocker<br>- Mineralocorticoid receptor antagonist<br>- calcium channel blocker<br>- thiazide or thiazide-like diuretic<br>- nitrates<br>- furosemide	ODB	Beta-blocker: if subclnam = "BETA-BLOCKERS"<br><br>Calcium channel blocker: if subclnam = "CALCIUM BLOCKERS" or "CALCIUM CHANNEL BLOCKERS"<br><br>Thiazide or thiazide-like diuretics: if subclan begins with DIURETIC and drugname is<br>- CHLORTHALIDONE<br>- HYDROCHLOROTHIAZIDE<br>- INDAPAMIDE<br>- AMILORIDE HCL & HYDROCHLOROTHIAZIDE<br>- SPIRONOLACTONE & HYDROCHLOROTHIAZIDE<br><br>Nitrates: drugname = "ISOSORBIDE DINITRATE" or "NITROGLYCERIN"<br><br>Furosemide: if drugname = "furosemide"	1-year lookback window from index	N (%)
Allopurinol	ODB	If drugname = "allopurinol"	1-year lookback window from index	N (%)
Methimazole	ODB	If drugname = "methimazole"	1-year lookback window from index	N (%)
Propylthiouracil	ODB	If drugname = "propylthiouracil"	1-year lookback window from index	N (%)
Statin	ODB	If subclnam in "STATINS" OR "CALCIUM BLOCKERS ANTILIPEMIC COMBINATIONS" (full name is ANTILIPEMIC: ATINS)	1-year lookback window from index	N (%)

Outcomes: Code Description

Outcome	Data Source	Variable	Time	Reporting Detail
ANCA Vasculitis	DAD<br>NACRS	Large vessel vasculitis: M35.3 M31.5 M31.6 M31.4	After index date	N (%)

© 2026 Fremont D et al. *JAMA Network Open*.

		Medium/small vessel vasculitis: M313 M313.1 M317 M301 M31.0 M30.0 M30.1 L95.9<br><br>Other vasculitis: M31.1 M35.2 D69.0 I77.6 M30.3		
Secondary Outcome				
Positive Serology test	OLIS	Observation code: 6968-2 6969-0 8084-6 37992-5 8085-3 37991-7	After index date	N (%)

© 2026 Fremont D et al. *JAMA Network Open*.
