# Supplementary material for: Hydralazine Use and Risk of Vasculitis
Source: JAMA Netw Open. 2026 Mar 16;9(3):e261943. doi: 10.1001/jamanetworkopen.2026.1943 (PMC12993695; doi:10.1001/jamanetworkopen.2026.1943)
Supplement: Supplement 2. — Data Sharing Statement [file jamanetwopen-e261943-s002.pdf]

Data Sharing Statement

Fremont. Hydralazine Use and Risk of Vasculitis. *JAMA Netw Open*. Published March 16, 2026. doi:10.1001/jamanetworkopen.2026.1943

Data
Data available: Yes
Data types: Data dictionary
How to access data: Variables and datasets defined in supplementary materials.
When available: With publication

Supporting Documents
Document types: None

Additional Information
Who can access the data: N/A
Types of analyses: N/A
Mechanisms of data availability: N/A
